# Post-processing of biochars to enhance plant growth responses: a review and meta-analysis

**DOI:** 10.1007/s42773-021-00115-0

**Published:** 2021-08-25

**Authors:** Sean C. Thomas

**Affiliations:** Institute of Forestry and Conservation, University of Toronto, 33 Willcocks St., Toronto, ON M5S 3B3 Canada

**Keywords:** Activation, Granulation, Leachates, Particle size, Pelletization, Post-processing

## Abstract

A number of processes for post-production treatment of “raw” biochars, including leaching, aeration, grinding or sieving to reduce particle size, and chemical or steam activation, have been suggested as means to enhance biochar effectiveness in agriculture, forestry, and environmental restoration. Here, I review studies on post-production processing methods and their effects on biochar physio-chemical properties and present a meta-analysis of plant growth and yield responses to post-processed vs. “raw” biochars. Data from 23 studies provide a total of 112 comparisons of responses to processed vs. unprocessed biochars, and 103 comparisons allowing assessment of effects relative to biochar particle size; additional 8 published studies involving 32 comparisons provide data on effects of biochar leachates. Overall, post-processed biochars resulted in significantly increased average plant growth responses 14% above those observed with unprocessed biochar. This overall effect was driven by plant growth responses to reduced biochar particle size, and heating/aeration treatments. The assessment of biochar effects by particle size indicates a peak at a particle size of 0.5–1.0 mm. Biochar leachate treatments showed very high heterogeneity among studies and no average growth benefit. I conclude that physiochemical post-processing of biochar offers substantial additional agronomic benefits compared to the use of unprocessed biochar. Further research on post-production treatments effects will be important for biochar utilization to maximize benefits to carbon sequestration and system productivity in agriculture, forestry, and environmental restoration.

**Supplementary Information:**

The online version contains supplementary material available at 10.1007/s42773-021-00115-0.

## Introduction

Pyrolyzed organic matter, particularly which derived from waste material streams from forestry and agriculture, has received considerable recent attention for a wide range of applications. When applied to soils this material has been labeled “biochar” (Lehmann and Joseph 2015). Applications in agriculture continue to be a main focus of research; however, biochar utilization in ecological restoration and land reclamation (Beesley et al. 2011), and forestry (Thomas and Gale 2015), has also increased. In addition, biochar is increasingly in use for non-soil applications, such as water filtration (Mohan et al. 2014), in animal feed (Joseph et al. 2015a, b), as a product substitute for fossil-fuel-derived components used in composite materials (e.g., Peterson 2013), and in high-tech applications such as supercapacitors (Jiang et al. 2013). The prevalent definition of biochar is “charred organic matter…applied to soil in a deliberate manner, with the intent to improve soil properties” (Lehmann and Joseph 2015, p. 2). Thus, non-soil uses might be better described by the term “bio-carbon” (retaining “charcoal” for fuel uses), but the terminology remains unsettled (Hagemann et al. 2018). There is also considerable potential for multiple use strategies, such as biochar use in animal feed followed by agricultural utilization of biochar-enriched manures. Regardless of the specific end-use, the material properties of biochar, in particular its high porosity, sorptive capacity, and recalcitrance to mineralization, as well as its overall ecological sustainability, commonly offer considerable advantages over other materials.

A main motivation for recent attention to biochar–bioenergy systems is their potential to have a net carbon-negative impact: i.e., to result in net withdrawal of CO_2_ from the atmosphere (Woolf et al. 2010; National Academies of Sciences, Engineering, and Medicine 2018). The net carbon balance of such a system depends on a number of factors, with the principle being: (1) baseline emissions of feedstock materials if not used in biochar production; (2) the efficiency of bioenergy produced and its distribution; (3) the degree to which biochar can act as a substitute for carbon-emitting technologies; (4) the carbon footprint of the feedstock supply chain and processing; (5) the effects of biochar on net carbon uptake in managed ecosystems. This fifth factor is often critical to achieve long-term carbon-negative impacts, since photosynthesis remains one of the only processes that can be harnessed on a large scale to directly take up atmospheric CO_2_. Many managed ecosystems show nutrient and/or water limitation of net ecosystem productivity, particularly in degraded ecosystems or systems with naturally low soil nutrient status. The potential for a positive feedback loop—in which production and application of biochar ultimately yields additional capacity to produce biochar—is contingent on biochar applications having positive effects on net productivity and carbon balance of managed ecosystems where it is deployed as a soil amendment.

Biochar producers, ranging from large-scale industrial operations to those at the farm or garden scale, commonly perform post-processing steps intended to improve biochar performance. Perhaps the most common example of biochar post-processing in the nascent biochar industry, and in small-scale operations, is manipulation of particle size. Grinding and/or sieving of raw biochars is common to increase product uniformity and to reduce particle size under the assumption that fine particles will have better mixing properties with soil. Conversely, increasing particle size through pelletization or granulation is also done in an effort to reduce wind transport of fine particles during and after biochar applications. The relative benefits of increasing or decreasing particle size of biochars are not obvious. For example, biochar particle size effects on soil water-retention capacity have varied among studies, with higher values for small particle sizes in some studies (Zhang et al. 2010), and the reverse pattern in others (Chen et al. 2017). It has been suggested that these effects may differ qualitatively depending on the particle size distribution of the soil in question (Liu et al. 2016). In one of the few studies to experimentally examine growth responses to biochar particle size across species, one plant species showed a higher growth response to large particles, while a second showed a higher growth response to small particles (Liao and Thomas 2019).

A wide variety of post-processing methods other than manipulations of particle size have also been employed. Steam activation has long been used to enhance surface area and porosity characteristics of activated carbons, while removing mobile organic and inorganic constituents (Sizmur et al. 2017). Steam activation is regarded as generally being too expensive an option for biochar production, and recent research has focused on chemical oxidation as a means of achieving similar benefits (e.g., Huff and Lee 2016). Chemical oxidation also mimics some aspects of biochar ageing and weathering that occur in situ, which have been associated with soil benefits in a number of studies. The importance of physical post-processing of biochar has been highlighted as a practical matter in the industry (e.g., Marrero 2018); however, reviews addressing biochar post-processing have focused on water treatment and metals sorption applications (Yavari et al. 2015; Hagemann et al. 2018; Sajjadi et al. 2019).

The purpose of the present paper is first to review available published literature on physical post-processing of biochars for use as a soil amendment, providing an overview of processing effects on physio-chemical properties of biochars from a “plant’s eye” perspective, and second, to formally assess effects of biochar post-processing on plant growth using meta-analysis techniques. Excluded from the review are all forms of soil co-amendments (e.g., compost, fertilizers) and biological post-processing methods (e.g., co-composting, inoculation). Also, excluded are “doping” methods involving additions of metals to biochars (e.g., Joseph et al. 2015a, b). The post-processing treatment that has received the most attention is manipulation of biochar particle size: I therefore also examine available data on particle-size-specific effects on plant growth. In addition, post-pyrolysis water quenching of biochar is in widespread use, and this brings up the important consideration of the chemistry and potential valorization of the leachate derived from this process. The literature on biochar leachate effects is thus also reviewed, and the effects of post-production biochar leachates on plant growth performance are evaluated.

### Overview and classification of post-processing methods for biochar

A provisional classification of post-treatment biochar processing methods is presented in Table 1: (i) Water quenching or leaching is mainly intended to remove undesired substances from biochars, particularly organic molecules that can condense on biochars during cooling. These condensed chemicals can include toxic organics, but also a wide range of chemicals that effectively clog biochar pores or reduce mixing between the soil solution and biochars. (ii) Heating and aeration treatments may have similar effects to leaching but are expected to only remove volatile compounds as well as sorbed gases such as ethylene. (iii) Ageing and weathering in soils enhance many biochar properties through surface oxidation and increased porosity, suggesting post-processing treatments to mimic these effects. (iv) Activation treatments are generally intended to increase porosity characteristics and surface functional groups but may also result in similar effects to leaching treatments. (v) Reductions in biochar particle size are motivated by enhanced particle mixing within soils, and potentially enhancing porosity and hydrological properties. (vi) Biochar pelletization/granulation is unique in that it has been proposed mainly as a means of reducing generation of biochar dust particles and to increase ease of handling but has not generally been presumed to have agronomic benefits in terms of plant growth and yield. In the following sections, I review in greater detail available information on each of these treatments, and currently available information on how each affects physio-chemical properties of biochars as they are related to agronomic uses.

**Table 1:** Classification of physio-chemical post-processing methods for biochar based on the primary mechanisms for enhancing property characteristics

Treatment type	Rationale/proposed mechanism(s)
Water quenching and leaching	Removal or reduction in mobile organic compounds sorbed during pyrolysis; reduction in ash fraction; increased surface area due to unblocking of pores
Heat treatments/aeration	Similar benefits as cleaning/activation treatments, while retaining mineral nutrients
Ageing/weathering	Increase in O-containing surface functional groups; increased porosity; reduced pH
Activation	Increased surface area and pore volume, plus similar benefits as leaching treatments
Particle size reduction	Increased particle mixing within soils
Pelletization/granulation	Dust reduction; increased ease of handling; reduced erosion and wind transport

### Water quenching and leaching

Biochars are commonly “quenched” with water at the end of the pyrolysis process. This is a necessary step to terminate pyrolysis in a variety of low-tech systems, such as conical kiln “flame-curtain” pyrolyzers (Cornelissen et al. 2016; Page-Dumroese et al. 2017). Water quenching immediately following production is also described as an essential step to inhibit combustion in many retort kilns (e.g., Shepard 2011), and medium-scale mobile pyrolysis systems (e.g., Archuleta and Page-Dumroese 2019). Similar measures are commonly employed in large-scale biochar production facilities, with additions of water eliminating the risk of spontaneous combustion, cooling the material to ease handling, and controlling biochar dust. Following application, biochars are also generally subject to repeated leaching in the natural soil environment following rainfall events.

Water quenching has the potential to remove readily solubilized components from biochar. There is now a somewhat extensive literature characterizing the chemistry of biochar leachates (e.g., Spokas et al. 2011; Mukherjee and Zimmerman 2013; Buss et al. 2015; Lievens et al. 2015; Liu et al. 2015; Rombolà et al. 2015; Gale et al. 2016; Wang et al. 2017a, b). Compounds that are not strongly bound to the biochar matrix, and that are water-soluble, are likely to be dissolved in the leachate generated by post-production quenching. Such compounds include soluble minerals, such as potassium and sodium, wood vinegar constituents, such as acetic acid and phenol, and other water-soluble organics present in condensed forms on the surfaces of biochars. Poly-aromatic hydrocarbons (PAHs) of concern as persistent organic pollutants have received the most attention as toxic constituents in biochar leachates (Quilliam et al. 2013; Buss and Mašek 2014; Wang et al. 2017a, b). Another biochar constituent expected to be reduced or removed through leaching is inorganic carbon in the form of carbonates, a major constituent of ash (Gaskin et al. 2008).

Leachates obtained from freshly produced biochar may not correspond precisely to those obtained from biochar quenched as a means to halt the pyrolysis process or immediately post production. Cornelissen et al. (2016) compared a total of 17 biochars made with several designs of simple open flame-curtain (“Kon–Tiki”) kilns: two designs were quenched with water and two with soil. Water-quenched and soil-quenched biochars were similar in terms of BET surface area and PAH levels, but water-quenched biochars showed higher cation exchange capacity (CEC). Water quenching was also associated with reduced carbon yields, likely reflecting losses of carbonates and mobile organic compounds. Although providing the only available direct information on post-pyrolysis quenching, this particular study confounds differences in kiln design with quenching treatments. Comparable studies on other pyrolysis systems are needed.

### Biochar leachates

Leaching treatments for biochar raise the issue of the chemistry of biochar leachates and their potential for valorization as a fertilizer or soil amendment. There are strongly divergent perspectives in the literature on the potential value of leachates in this regard. One body of research has suggested that dissolved organics in biochar leachates may promote plant growth (Lou et al. 2016; Yuan et al. 2017; Bian et al. 2019). In contrast, other studies have found strongly negative effects of biochar leachates on plant growth performance (e.g., Buss and Mašek 2014; Gale et al. 2016), and acute toxic effects of biochar leachates have been demonstrated in lab assays for both higher plants (e.g., Rogovska et al. 2012), and other organisms, including bacteria, protozoa, algae, and crustaceans (Oleszczuk et al. 2013; Bastos et al. 2014). Most available data pertain to seed germination and early seedling development, which likewise indicate a range of both positive effects (Sujeeun and Thomas 2017; Gezahegn et al. 2021) and, more commonly, negative effects (Rogovska et al. 2012; Buss and Mašek 2014; Roberts et al. 2015; Rombolà et al. 2015; Wang et al. 2017a, b; Alshahrani and Suansa 2020). Although leachates from a wide range of chars can show phytotoxicity, germination inhibition appears to be particularly acute for leachates from hydrochars (Fornes and Belda 2017; Puccini et al. 2018), and fast-pyrolysis biochars (Gezahegn et al. 2021).

Elucidating the mechanisms for either phytotoxicity or putative beneficial effects of biochar leachates is complicated by the wide variety of organic and inorganic compounds found in leachates, by high variability among biochars, and by species-specific responses. There is evidence that biochars with high-volatile organic compound concentrations tend to show higher phyto-toxicity (Buss and Mašek 2014). Although PAHs have received most attention as potentially toxic constituents, higher concentrations of volatile fatty acids (such as acetic and propionic acid) and phenols that are the main constituents of wood vinegar are common in biochars (Buss et al. 2015; Gale et al. 2016; Gezahegn et al. 2021). These compounds are recognized to commonly be phytotoxic and to have high solubility (Buss et al. 2015; Rombolà et al. 2015; Gale et al. 2016). In addition, some biochars have high concentrations of soluble phytotoxic metals inherited from feedstock material (e.g., Huang et al. 2018; Phoungthong et al. 2018). Very high pH or excessively high potassium concentrations may also inhibit seed germination and early plant development (Buss et al. 2016).

Positive effects of biochar leachates on germination and seedling development, as observed in a number of studies (e.g., Sujeeun and Thomas 2017; Gezahegn et al. 2021), could likewise potentially be related to either inorganic or organic constituents. Most biochars are high in potassium, which is highly soluble in ionic form, and which can promote seed germination and early seedling development (Bewley et al. 2013; Sher et al. 2019); high pH, attributable to soluble alkaline elements and carbonates, may also enhance early seedling performance (e.g., Olsson and Kellner 2002). It is, however, plausible that biochars also contain organic molecules that stimulate early plant development and growth; in particular, Karrikins constitute a likely group of molecules known to act as plant hormones that are apparently common in trace quantities in biochars (Kochanek et al. 2016). However, studies purporting to demonstrate bio-stimulation of early plant development by organic molecules in biochar leachates or extracts (e.g., Sun et al. 2017; Yuan et al. 2017; Bento et al. 2020) have generally not included controls that rigorously exclude effects of inorganic nutrients or solution pH. It is also critical to note that many substances that promote seed germination by degrading the seed coat or leaching inhibitors may be detrimental to later stages of plant growth and development. In addition, biochar effects on germination and early seedling development under field conditions may deviate strongly from lab trials: for example, in forest systems, phenolics in the litter layer often strongly inhibit seedling development, and accelerated seedling development may mainly be a response to biochar sorption of these inhibitors (Thomas 2021).

### Heat treatment and aeration

Some post-processing of biochar is essentially inevitable, even if mostly passive, such as aeration by exposure to ambient air following pyrolysis. Freshly produced biochars can pose a risk of spontaneous combustion mainly attributable to volatile organic compounds produced during pyrolysis (Zhao et al. 2014). Leaching of biochars is likely to remove both substances beneficial to plant growth, such as highly mobile potassium ions, and inhibitory, such as volatile fatty acids. An alternative “cleaning” process for biochars is heat treatment to selectively remove organic volatiles, generally combined with aeration. In addition to liquid-phase molecules, freshly produced biochars also contain sorbed or physically occluded gas-phase constituents, such as the plant hormone ethylene (Spokas et al. 2010). Simple post-production aeration of biochar has been suggested as a means to off-gas ethylene (Fulton et al. 2013). Napthalene is generally the most abundant polyaromatic hydrocarbon of toxicity concern that is detected on biochars (Fabbri et al. 2013); this compound is highly volatile but has low solubility in water, and thus is expected to be removed by heat treatment and aeration, but not water quenching or leaching. Many other organic molecules produced during pyrolysis are likely to be sorbed on the biochar surface, and would similarly have low solubility, but sufficient volatility to be removed through heating and aeration treatments (Buss and Mašek 2016). Reductions or complete removal of volatile organic compounds and associated reductions in toxicity have been documented in several studies (Buss and Mašek 2016; Kołtowski and Oleszczuk 2015; Gale et al. 2016; Intani et al. 2019). However, effects of heating and aeration treatments on biochar structure (e.g., increased surface and porosity due to removal of compounds occluding pores) and chemistry (e.g., surface oxidation) have received little research attention.

### Ageing and weathering

Changes in the properties of biochars, and the consequences of these changes to net effects on crop growth and yield, are obviously a critical issue in most field applications of biochar. There is accordingly a large literature focused on the duration of biochar effects on soil properties and crop productivity through multiple cropping cycles. A common pattern noted in many studies is that biochar amendments can have neutral or negative effects in the first year of application, but positive effects in subsequent years (e.g., Major et al. 2010; Basalirwa et al. 2019; Dong et al. 2019; Manzoor et al. 2019). There is evidence from a variety of studies that ageing and/or weathering of biochars in situ can improve biochar properties. For example, aged biochars can show increases in both cation exchange capacity (CEC) (Liang et al. 2006), and capacity to sorb and immobilize heavy metals (Uchimiya et al. 2010a, b; Bakshi et al. 2014), likely due to increases in oxygen-containing functional groups associated with oxidation processes in the soil. There is also some evidence that aged biochars show stronger effects in terms of reducing soil greenhouse gas emissions (Mukherjee and Lal 2013).

Many of the physio-chemical changes in biochar associated with leaching and aeration are likely to also be associated with ageing and weathering processes, particularly after biochar is applied to soils, or when biochar is stored in an open setting exposed to the elements. However, natural weathering processes are also likely to result in a greater extent of surface oxidation (Mia et al. 2017). Concurrent with surface oxidation and leaching, naturally weathered biochars can also become quite acidic: e.g., naturally aged fire residue charcoals used in experiments have had pH values of 3.9 (Esfandbod et al. 2017) to 3.1 (Rashti et al. 2019). In addition, biochars interact closely with clay particles in the soil, forming biochar-mineral complexes (Joseph et al. 2010). Another potentially important interaction is the immobilization of hydrophobic dissolved organic carbon by complexation with polyvalent metal ions in the soil (Gámiz et al. 2019). Finally, ageing and weathering in situ are likely strongly influenced by both microbial processes and freeze–thaw cycles.

Relatively little attention has been paid to intentional ageing or weathering of biochar as a potential post-processing step; however, several lines of evidence suggest this could be beneficial. Mukherjee et al. (2014) compared physiochemical properties of a biochar kept from contact with soil with a mesh bag, but exposed to air, leaching, temperature variation, and microbes over 15 months, to the same biochar incubated in soil and to the freshly produced biochar. The field-aged biochar showed fivefold increases in CEC, increases in O-containing surface functional groups, the appearance of detectable anion exchange capacity (AEC), and reduced pH and ash content (Mukherjee et al. 2014). Similar results have been obtained in some subsequent studies examining pre-application biochar ageing (Heitkötter and Marschner 2015; Xu et al. 2018), although one other mesh bag ageing experiment did not find large changes in biochar properties (Gámiz et al. 2019). Xu et al. (2018) found that simulated ageing involving freeze–thaw and rewetting cycles reduced biochar pH and increased O-containing functional groups more so than a similar period of ageing in situ. A recent experimental inoculation study suggests that microbial activity, rather than abiotic processes, may be mainly responsible for this oxidation (Quan et al. 2020).

### Activation treatments

Could the benefits of ageing and weathering in situ be realized by physio-chemical post-processing of biochars? The most common methods used experimentally to simulate natural biochar ageing have been chemical oxidation using strong oxidizing agents such as H_2_O_2_, and, to a lesser degree, exposure to freeze–thaw cycles (Li et al. 2019a, b). In some cases, a combination of chemical treatments has been used to simulate both oxidation and acid exposure: for example, Bakshi et al. (2016) developed a procedure involving incubation of biochars with HCl and H_2_O_2_, followed by washing with CaCl_2_. In general, oxidation-based ageing methods do not precisely simulate natural ageing in the soil, and commonly result in a greater degree of surface oxidation that can destroy biochar pore structure and produce novel chemical products not found with natural ageing (Xu et al. 2018). Nevertheless, chemical oxidation is of particular interest as an economical and logistically simple post-processing treatment.

Hydrogen peroxide (H_2_O_2_) is the oxidizing agent that has been most commonly applied to biochar post-processing for soil applications (Lee et al. 2013; Huff and Lee 2016; Mia et al. 2018, 2019; Paymenah et al. 2018). An early paper by Xue et al. (2012) found that H_2_O_2_ treatment of hydrochar enhanced heavy metal sorption associated with O-containing functional groups; similar results have been obtained in other studies focused on toxic metals sorption by biochars (Zuo et al. 2016; Wang and Liu 2018; Xia et al. 2019). Beyond increases in surface oxidation, H_2_O_2_ treatment can reduce biochar pH and increase specific surface area and CEC (Lee et al. 2013; Huff and Lee 2016; Huff et al. 2018), though decreases in CEC have also been reported in some cases (Vaughn et al. 2017), as have reductions in BET surface area (Vaughn et al. 2017). H_2_O_2_ treatment also generally removes inorganic mineral components from the biochar matrix (Sizmur et al. 2017), which is unlikely to be beneficial to plant growth.

A wide variety of acid and base treatments have also been explored for chemical modification of biochars, but typically with an emphasis on water treatment and engineering applications (Sizmur et al. 2017; Hageman et al. 2018; Sajjadi et al. 2019), rather than soil applications. Treatments with bases (typically KOH, NaOH, or K_2_CO_3_) can enhance BET surface area (e.g., Azargohar and Dalai 2008). Acid treatments of biochar for soil applications (generally using nitric, sulfuric, phosphoric, or oxalic acids) have been motivated as a means to increase biochar pH and remove mobile organics, and have been found effective in both respects (e.g., Fornes and Belda 2017, 2019; Vaughn et al. 2017)). Acid treatments can also increase both BET surface area and surface oxidation, though treatment conditions strongly influence results (e.g., Yorgun and Yıldız 2015).

Steam treatment, with or without presence of CO_2_, has a long history of use to produce activated carbons, but has generally been replaced by chemical processes for commercial activated carbon production (Yargicoglu et al. 2015; Hilber et al. 2017; Lee et al. 2018). Steam activation has thus not commonly been investigated in terms of its effects on biochar structure or effects as a soil amendment. Steam treatment typically substantially increases BET surface area (Azargohar and Dalai 2008; Lima et al. 2010; Uchimiya et al. 2010a; Borchard et al. 2012) but can also increase ash content and pH (Borchard et al. 2012); available data suggest that steam activation also increases CEC (Borchard et al. 2012). Steam activation generally greatly reduces mineral nutrient content of biochars (Rezende et al. 2016); it may also enhance surface oxidation, depending on process temperature and the gas-phase environment during the process (Hagemann et al. 2018). There are few data available examining effects of steam-activated biochar on plant performance (Borchard et al. 2012; Rezende et al. 2016).

### Grinding, sieving, and particle sorting

Many important mechanisms for positive biochar effects, including retention and provision of plant nutrients, liming, sorption of toxic compounds, and beneficial effects on soil microbial communities, are contingent on adequate mixing of biochar and soil particles. In general, biochar will inherit the macrostructure and particle size distribution of the feedstock used, although fragmentation of particles commonly occurs (Downie et al. 2009; Spokas et al. 2014). Particle fusion during pyrolysis may also occur but appears to only be important under high pressures (Cetin et al. 2004); following soil application, particle agglomeration is, however, common due to biochar–clay interactions (Khademalrasoul et al. 2014). Wood chips or coarse sawdust are common forestry-related feedstocks; common agricultural feedstocks include rice hulls, grass stalks, corncobs, and bagasses. Very large feedstock (e.g., logging “slash” consisting of whole branches and other non-merchantable wood) is suitable as input for open kiln and flame-curtain pyrolysis systems. Smaller-sized feedstock may result in raw biochars suitable for direct use, but in many cases some processing to reduce particle size is necessary. Biochars generally have low mechanical strength and are highly friable (Downie et al. 2009); thus, some proportion of dust-sized particles are typically generated during pyrolysis, handling, and application (Ravi et al. 2016).

Many physio-chemical properties of biochars are strongly dependent on particle size (Table 2). Sangani et al. (2020) recently compared a wide variety of properties across size classes of sieved biochars derived from several feedstock sources and found that in general physical properties were strongly influenced by particle size, while feedstock effects were more important for chemical properties. Particle size has particularly strong effects on porosity characteristics, such as total porosity, mean pore diameter, and BET surface area (Sangani et al. 2020). Smaller particle size is also commonly associated with increased water-retention capacity (Lim et al. 2017; Liao and Thomas 2019; Sangani et al. 2020). However, in the soil matrix, small biochar particles can reduce water-retention capacity by filling soil inter-pores (Liu et al. 2016; Lim et al. 2017); hydraulic conductivity can likewise be reduced (Esmaeelnejad et al. 2017; Ibrahim et al. 2017). Some physical properties of biochars that are not strongly affected by particle size, or show variable results, include water repellency and skeletal density (Table 2). Responses of soil–biochar mixtures to biochar particle size generally parallel those for isolated biochar samples (Table 2). Particle size of biochar may also strongly influence soil particle cohesion and erosion potential (Li et al. 2019a, b), but this has received less attention than hydraulic properties.

**Table 2:** Selected physical and chemical properties of biochar as a function of particle size

Property	Direction of effect	References*
Physical properties of biochar		
Bulk density (g/cm^3^)	Fine > coarse	(1)
Specific surface area (m^2^/g)	Fine > coarse	(2–8)
Skeletal density (g/cm^3^)	No effect	(3)
Macro-porosity (%)	Coarse > fine	(2)
Mean pore diameter (nm)	Coarse > fine	(2,9)
Water repellency	Variable	(2)
Water retention capacity	Variable	(1,3,4,8,9)
Chemical properties of biochar		
EC	Fine > coarse	(2,9)
pH	Fine > coarse	(1,2,3,9)
Ash content	Variable	(1,2)
CEC	Fine > coarse	(2)
AEC	Fine > coarse	(2)
Physical properties in soil mixture		
Bulk density	Fine > coarse	(11,12)
Total porosity	Coarse > fine	(11,12,13)
Water retention capacity	Fine > coarse	(4,11,13)
Hydraulic conductivity	Coarse > fine	(3,10,11)
Soil shear strength	Fine > coarse	(14)
Soil compressibility	Fine > coarse	(14)
Chemical properties in soil mixture		
EC	Variable	(1,2,15)
pH	Fine > coarse	(1,2,8)
Time to equilibrium pH	Fine > coarse	(2,8)

*References—1: Liao and Thomas 2019; 2: Chen et al. 2017; 3: Sangani et al. 2020; 4: Esmaeelnejad et al. 2017; 5: Ibrahim et al. 2017; 6: Zhang et al. 2010; 7: Sun et al. 2012; 8: Rees et al. 2014; 9: Lebrun et al. 2018; 10: Liu et al. 2016; 11: Głąb et al. 2016; 12: Obia et al. 2016; 13: de Jesus Duarte et al. 2019; 14: Reddy et al. 2015; 15:Lu et al. 2014

Particle size can also have substantial effects on biochar chemical properties (Table 2). Smaller particle size feedstock material will generally undergo more complete pyrolysis, associated with greater losses of volatile materials, loss of O-containing functional groups, and increased concentration of alkaline elements—which result in increased ash content and pH. There is also evidence for increased CEC and AEC in biochars derived from small particles (Sangani et al. 2020), though data are surprisingly lacking on this point for biochar–soil mixtures. Smaller particles more readily allow escape of volatile organics during pyrolysis, potentially mitigating toxic effects (Dutta et al. 2017). Biochar feedstocks are generally variable in particle size, and thus particle sizes of sieved biochars are likely to reflect particle size pertaining during pyrolysis. Reductions in particle size by sieving processes are thus more likely to result in differences in chemical properties than grinding (Liao and Thomas 2019).

In addition to size, biochar particle geometry can importantly affect packing with soil particles, with non-spherical geometries acting to strongly enhance particle inter-pores (Liu et al. 2017; Yang et al. 2018; Liao and Thomas 2019). Mechanical sieving will generally allow relatively long, slender particles to pass through the sieving process, enhancing aspect ratio (Liao and Thomas 2019). Grinding vs. sieving processes may thus have quite distinct effects on physical properties of biochars, with grinding producing more spherical geometries.

At the extreme of small particle size, a number of studies have examined properties of biochar nanoparticles (i.e., with dimensions of 1–100 nm). Nanoparticles may be extracted from biochars through use of aqueous suspensions alone or with sonication treatments (e.g., Saxena et al. 2014; Liu et al. 2018), through high-energy ball-milling (e.g., Wang et al. 2018), or through a combination of techniques. A few studies have presented evidence for positive effects on biochar nanoparticles on seedling development (Saxena et al. 2014; Yue et al. 2019; Shen et al. 2020), but studies beyond the seedling stage do not appear to have been undertaken and mechanisms specific to nanoparticles remain speculative. Biochar nanoparticles may be formed in situ through physical fragmentation, solubilisation, and chemical oxidation, and thus play a role in long-term effects of biochars on soil properties (Bird et al. 2015).

### Pelletization and granulation

Depending on feedstock, handling, and pyrolysis methods, biochars are commonly produced with a high proportion of fine particles < 10 µm (Ravi et al. 2016; Li et al. 2018). These fine particles are easily transported by wind, and thus are likely to contribute to product losses during field applications. Fine biochar particles also constitute a potential human health hazard, particularly for personnel involved in handling and applying materials (Gelardi et al. 2019). Wind and water erosion mitigation is an important issue in biochar uses for environmental restoration (Kuttner and Thomas 2017; Li et al. 2019a, b). In addition, particulate matter emissions related to biochar utilization are of concern from a climate change perspective, as black carbon in the atmospheric dust cycle is an important radiative forcing agent (Ravi et al. 2016). These considerations have been the main motivation for research on biochar pelletization and granulation, as well as efforts along these lines within the nascent biochar industry.

Pellets are produced by compression, usually involving the forcible extrusion of materials through a die. Biochar pellets can generally be produced using standard die pelletizers even in the absence of a binding agent, but such pellets show very low mechanical strength (Hu et al. 2015; Al-Zayat 2017), mechanical strength being necessary to ensure that pellets remain intact during transportation and application. A variety of binding agents have been used successfully in biochar pelletization (Table 3). These include both organic materials (Kraft and alkaline lignin, starch, and pig manure compost), and chemical agents (e.g., polylactic acid, Ca(OH)_2_, and NaOH). In contrast to pelletization, granules are produced by an agglomeration process, generally using application of liquids fed as a mist onto seeding particles. Some binding agent is necessary in any granulation process. Binding agents that have been used in biochar granulation include hydroxypropyl methylcellulose, sodium carboxymethyl cellulose, starch, molasses, and fine lake sediments (Table 3).

**Table 3:** Summary of published studies on pelletization and granulation of biochar

Method/binding agent	Biochar feedstock	Proportion binder	Aggregate dimension	Compressive strength (MPa)	Reference*
Drum/pan granulation					
Hydroxypropyl methylcellulose	Cornstalk	3–9%	1–4 mm	0.15–0.50	(1)
Hydroxypropyl methylcellulose	Cornstalk	3–9%	1–4 mm	0.15–0.50	(2)
Hydroxypropyl methylcellulose	Birch bark	3–9%	1–4 mm	0.15–0.50	(2)
Hydroxypropyl methylcellulose	Miscanthus	3–9%	1–4 mm	0.15–0.50	(2)
Sodium carboxymethyl cellulose	*Prosopis juliflora*	5–14%	1–5 mm	“comparable to commercial fertilizer granules”	(3)
Starch	*Prosopis juliflora*	11–18%	1–5 mm		(3)
Hydroxypropyl methylcellulose	Birch bark	3–9%	1–4 mm (lowest yield)	> 96% attrition resistance	(4)
Molasses	Birch bark	20–40%	1–4 mm	> 98% attrition resistance	(4)
Ammonium nitrate	Birch bark	20–40%	1–4 mm (best yield)	> 94% attrition resistance	(4)
Lake sediments	Wood	23%	3–8 mm	0.19–0.21	(5)
Die pelletization					
Polylactic acid and starch	Mixed	7%/7%	4.8 mm	NA	(6)
No binder	Rice husk	0%	20 mm	0.65	(7)
Alkaline lignin	Rice husk	5–20%	20 mm	2.8–3.8	(7)
Starch	Rice husk	5–20%	20 mm	0.5–1.0	(7)
Calcium hydroxide	Rice husk	5–20%	20 mm	3.8–5.6	(7)
Sodium hydroxide	Rice husk	5–20%	20 mm	11.1–16.8	(7)
Kraft lignin	*Acer saccharum*	5–25%	2 mm	NA	(8)
Pig manure compost	Rice hull	10–80%	0.5 mm	NA	(9)
Kraft lignin	*Panicum virgatum*	10–30%	6 mm	2.3–4.7	(10)

*References—1: Bowden-Green and Briens 2016; 2: Briens and Bowden-Green 2019; 3: Reddy et al. 2018; 4: Briens and Bowden-Green 2020; 5: Vincevica-Gaile et al. 2019; 6: Dumroese et al. 2011; 7: Hu et al. 2015; 8: Al-Zayat 2017; 9: Shin et al. 2018; 10: Kim et al. 2014

*NA* not available

Recent published studies on biochar pelletization and granulation have focused on engineering of processes and mechanical properties of the aggregates formed (Hu et al. 2015; Bowden-Green and Briens 2016; Briens and Bowden-Green 2019, 2020; Reddy et al. 2018). Pelletized biochar has been examined as a seed coating (Williams et al. 2016), and as a component in plant nursery substrates (Dumroese et al. 2011; Andrenelli et al. 2016); effects on soil hydrological properties have also been investigated (Andrenelli et al. 2016). However, there is presently little information available on effects of biochar pelletization/granulation on biochar physio-chemical properties, though compression of macro-pores in pellets is apparent from electron micrographs (Al-Zayat 2017). One obvious general prediction regarding biochar pellets or granules is that such aggregates would generally show properties similar to larger-sized biochar particles (i.e., the differences noted in Table 2). However, while pellets may be generated using relatively coarse biochars, granule formation requires milling of biochar to a powder prior to granulation. In addition, binding agents may have substantial effects on soil processes and plant responses that are distinct from aggregation effects. I am not aware of any published study on biochar granules that has experimentally distinguished biochar aggregation effects from binding agent effects.

## Methods

Three sets of meta–analyses were conducted, one focused the effects of physio-chemical post-processing of biochar on plant growth, one on particle size effects, and one on the effects of biochar leachates on plant growth. In all cases, literature was searched using both ISI Web of Knowledge and Google Scholar, with a cutoff date of May 2020. ISI Web of Knowledge used explicit Boolean operators, while Google Scholar searches used proprietary AI-driven searches with combinations of 4–5 search terms, and a cutoff of 100 articles scanned per combination. In the case of searches related to post-treatment effects, search terms included descriptions for the set of terms in potential use to describe biochar (including “biochar”, “black carbon”, “char”, “charcoal”, “hydrochar”), in conjunction with a set of terms for plant growth (“plant” or agricult*” or “crop” or “tree” or “seedling” plus “growth”, “yield”, “biomass”, “performance”), and terms used to describe individual post-processing methods or the resulting products (“sieving”, “grinding”, “sieved”, “ground”, “nano”, “pelletization”, “pellet”, “granulation”, “granule”, “leaching”, “leached”, “washing”, “washed”, “ageing”, “aging”, “aged”, “weathering”, “weathered”, “heat-treatment”, “activation”, “acid-treatment”, “acidification”, “acidulation”, “base-treatment”, “basification”). In the case of searches related to biochar leachate effects, search terms included above set of biochar and plant growth terms, plus terms for leachate (“leachate”, “leached”, “filtrate”). Titles and abstracts were initially scanned for articles that would plausibly present original data on plant growth responses, and tables and figures of those articles searched for usable data. Articles presenting usable data were themselves read for citations to related articles, and citations to those articles were likewise systematically searched for usable data. Data from unpublished theses not otherwise available were included, though these represent a small proportion of the dataset.

Meta-analyses utilized the response ratio (*R* = ln(*X*_t_/*X*_c_) as the effect size statistic, where *R* is the response ratio statistic, *X*_t_ is the treatment mean and *X*_c_ is the control mean; pooled *R* values inversely were weighted by sampling variance. The response ratio is the most widely utilized meta-analysis effect size metric appropriate for mixed effects models where variation among studies is assumed to reflect both sampling error and random variation and is also the prevalent effect size metric used in prior analyses of biochar effects (e.g., Jeffery et al. 2011; Biederman and Harpole 2013; Thomas and Gale 2015; Wang et al. 2016). Data were extracted from tables (or original data) where possible; graphical data were digitized using webplotdigitizer (Rohatgi 2019). Total biomass responses were used as the plant growth metric where available. Alternative growth measures used included aboveground biomass, fresh mass, agronomic crop yield, and percent cover, all of which scale linearly with total biomass. In the small number of cases where means were presented without error values, standard deviations were imputed from the observed average coefficient of variation observed across studies (Lajeunesse et al. 2013). To express response ratios as a percent change, the metric was back-transformed: i.e., percent change = 100 × (exp(R) − 1). Analyses were conducted in R version 3.3.3 (R Core Team 2017) using the metafor package (Viechtbauer 2010). Biochar and post-processing terms were treated as random effects and models were fitted by restricted maximal likelihood. Statistical tests for effects were based on the default normal approximation and the Q-test for heterogeneity (Viechtbauer 2010).

## Results

### Biochar post-treatment effects on plant growth

**Fig. 1:**
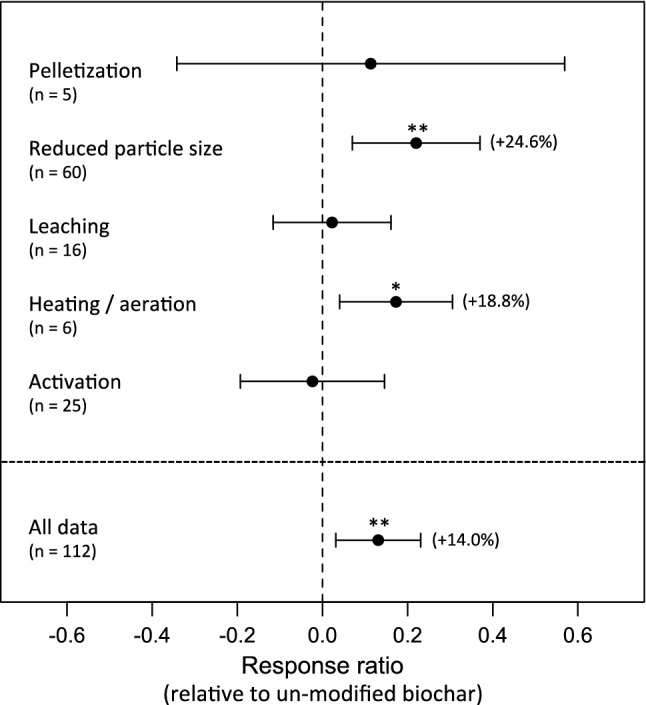
Meta-analysis of plant growth responses to biochar post-processing modifications, with response quantified relative to un-modified biochar. Response ratio statistics are shown ± 95% confidence limits

The physiochemical post-processing treatments of biochar examined generally did enhance plant growth responses (Fig. 1). The pooled response ratio statistic (*R* = 0.131 ± 0.051 SE) corresponds to an average 14.0% increase in plant biomass or yield above that reported for unmodified biochar. For the data considered here, the pooled response ratio statistic for unmodified biochars relative to no-biochar controls (*R* = 0.241 ± 0.056; *p* < 0.001) corresponds to a 27.3% increase in plant biomass or yield; for modified biochars relative to controls (*R* = 0.366 ± 0.072; *p* < 0.001), the proportional increase is 44.2%. In each of these analyses, the *Q*-test for heterogeneity among comparisons is significant (*p* < 0.001). In part, this variation is clearly attributable to post-processing type, which was found to be significant as a moderator of effects (*p* = 0.005); effects of other moderators examined (pot vs. field experiments; growth metric) were not significant. Reductions in particle size (*R* = 0.220 ± 0.076; *p* = 0.004), and heat/aeration treatments (*R* = 0.173 ± 0.068; *p* = 0.011) were the treatment types that resulted in significantly increased plant growth responses considered individually; pelletization, leaching, and activation treatments all were non-significant. In all data subsets, with exception of heating/aeration, the *Q*-test for heterogeneity among comparisons was significant (*p* < 0.05).

### Biochar particle size in relation to plant growth

**Fig. 2:**
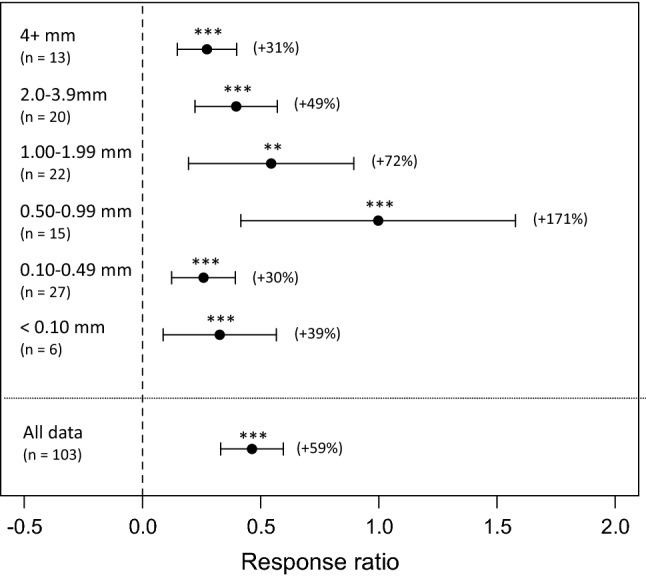
Meta-analysis of plant growth responses to variation in biochar particle size following grinding and/or sieving. Response ratio statistics are shown ± 95% confidence limits

In the preceding analysis, reductions in particle size showed the largest and most consistent enhancements in biochar effects on plant growth. The available data also permit an analysis of effects segregated by particle size (Fig. 2). All particle size classes showed significant positive effects on plant growth in comparison to controls, and particle size category was significant when considered as a moderator (*p* = 0.006). The response is clearly largest at the intermediate size classes examined: the 0.50–0.99 mm size class shows the highest response (*R* = 1.00 ± 0.30 SE), and 95% confidence limits for the response exceed the mean responses for both lower (< 0.5 mm) and higher (> 2 mm) particle size classes; R values for these size classes range from 0.24 to 0.33. The response for the 1.00–1.99 mm size class is intermediate (*R* = 0.54 ± 0.18), consistent with a unimodal response pattern with a peak somewhat less than 1 mm.

### Biochar leachate effects on plant growth

**Fig. 3:**
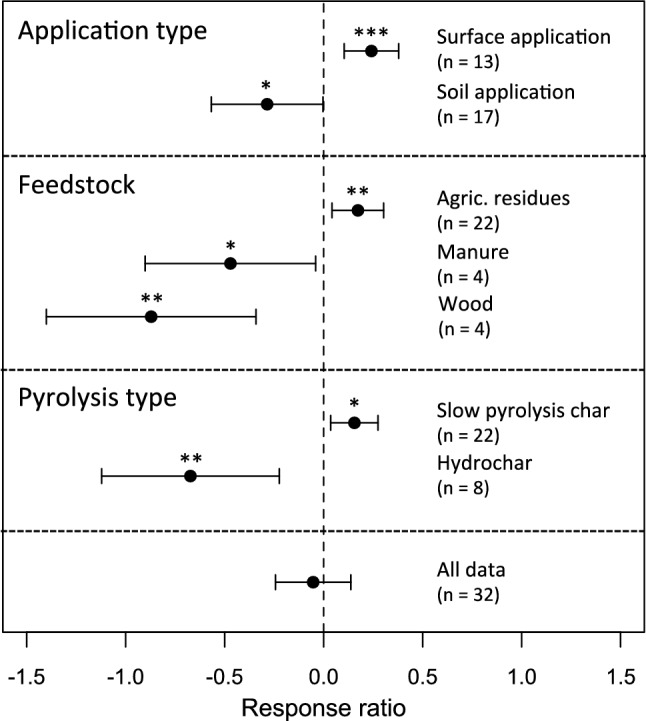
Meta-analysis of plant growth responses to additions of biochar leachates. Response ratio statistics are shown ± 95% confidence limits

As noted above, biochar leachates have been proposed to have both positive and negative effects on plant growth. The response ratio statistic pooled across all comparisons was not significantly different from zero (Fig. 3); however, this belies very large heterogeneity among comparisons that is likely explained by differences among biochar type, feedstock, and application methods (Fig. 3). Positive effects on plant growth were detected for slow pyrolysis chars, while negative effects were detected for hydrochars. Positive effects also detected for chars derived from agricultural residue feedstocks, while negative effects were detected for manure and wood-derived chars. In addition, positive effects corresponded to trials where leachates were applied as an aerosol to plant surfaces and/or the soil, while negative effects were found where leachates were added in liquid form to the soil (Fig. 3); the largest mean response ratios were specifically associated with these surface applications. These effects are largely confounded in the dataset: in particular, essentially all studies involving surface applications of biochar leachates have been conducted with slow pyrolysis biochar generated from agricultural residues, specifically wheat straws, rice husks, or maize stalks (Lou et al. 2016; Yuan et al. 2017; Bian et al. 2019). Although observed negative effects are in part attributable to hydrochars, there is evidence for negative effects of biochar leachates on plant growth from slow pyrolysis biochars as well (Gale et al. 2016).

## Discussion

The meta-analysis results indicate that post-processing of biochars can strongly enhance their effectiveness in promoting plant growth. In the dataset examined, the pooled response to unmodified biochar was ~ 27%, while post-processed biochar resulted in an additional ~ 14% growth enhancement on average. This overall effect was attributable mainly to two specific modifications that have large and predictable benefits to plant growth responses, namely processing to reduce particle size, and heat and/or aeration treatments to reduce volatile organics (Fig. 1). In contrast, leaching, activation, and pelletization/granulation did not result in additional statistically detectable plant growth benefits (Fig. 1). Compared to the overall available data on biochar effects on plant productivity (e.g., meta-analyses based on *N* = 371 (Biederman and Harpole 2013), and *N* = 450 (Liu et al. 2013) comparisons), *N* = 116 comparisons is a modest sample size. Nevertheless, the overall mean growth response to un-modified biochar is similar to (or somewhat larger than) these prior analyses. There is thus no indication of a bias toward experiments on modification of biochars that produced below-average growth responses, and that therefore might over-estimate the benefits of post-processing modifications.

Grinding and/or sieving to reduce biochar particle stands out as the modification that most reliably enhances biochar benefits to plant growth. However, the analysis of particle-size-specific effects (Fig. 2) suggests that it is not simply the case that “smaller is better”; rather, there appears to be an optimal particle size at a linear dimension of somewhat less than 1 mm. Although the prior literature on biochar particle size effects mentions the possibility of an optimal particle size (e.g., Liao and Thomas 2019), papers have generally emphasized the agronomic advantages of small particle sizes, such as enhanced mixing, increased liming effects and more rapid pH equilibration (e.g., Rees et al. 2014; Chen et al. 2017), increased CEC (He et al. 2018; Sangani et al. 2020), and increased micro-porosity (Sun et al. 2017). It is thus less clear what biochar properties would produce reduced growth responses at very low particle sizes. One possibility is effects on substrate porosity. Reduced biochar particle size is associated with reduced pore size and macro-porosity associated with disruption of larger pores (He et al. 2018; Sangani et al. 2020). Smaller biochar particles may also fill soil inter-pores, and thus result in reduced soil porosity (Zhang et al. 2010), though this also depends strongly on the soil particle size distribution and particle geometry (Liu et al. 2017; Liao and Thomas 2019).

The observation that biochar particle sizes of ~ 0.5–1.0 mm result is optimal in terms of effects on plant performance has immediate application in the nascent biochar industry. Milling of biochars to this specification is readily accomplished with conventional coarse grinders. It may also be beneficial to produce biochar pellets or granules in this approximate size range from very fine biochar residues. This particle size range approximates coarse sand and is less easily transportable by wind or water than finer particle sizes. Targeting production of this particle size range is therefore also likely to reduce negative effects of biochar dust in terms of human health (Gelardi et al. 2019), and potential adverse climatic feedbacks (Ravi et al. 2016). Limitations on this observation should, however, be noted. While the particle size meta-analysis does include some field observations, these derive from only 3 published papers (Billah et al. 2018, 2019; Manzoor et al. 2019) that only consider responses of leguminous crops in arid environments. In addition, no single published study on plant responses has considered more than three particle size categories. There is thus a clear need for additional studies that examine agronomic responses to a wide range of biochar particle sizes, and particularly for field studies to verify an optimal biochar particle size and better elucidate the mechanisms responsible for this pattern.

Both leaching treatments and heat treatment with aeration have been considered as means to reduce volatile organic compounds common on freshly produced biochars (e.g., Buss and Mašek 2016; Gale et al. 2016); the meta-analysis results strongly suggest that heating/aeration treatments produce consistently more favourable results in terms of plant growth responses than leaching treatments (Fig. 1). A likely explanation for this pattern is that leaching removes other biochar constituents favourable to plant growth, in particular readily solubilized mineral nutrients, such as K and P. Heat treatments may also be more effective in removing organic molecules that are volatile but not water-soluble, such as naphthalene. High variability in results from leaching treatments may be due to differences in dilution, biochar particle size, and specifics of temperature, duration and mixing characteristics during leaching.

It is also possible that leaching removes soluble organic compounds that are themselves beneficial to plant growth; however, the evidence as to whether this mechanism is plausible remains ambiguous. The meta-analysis presented here for biochar leachate effects essentially highlights high variability, with observed effects including both large positive and negative responses (Fig. 3). Moreover, there is a marked dichotomy in methodologies: studies that have recorded positive effects of biochar leachate applications on plant growth have utilized biochars generated by slow pyrolysis of specific agricultural waste materials feedstocks (corn stalks, wheat straws, or rice husks) and applied as a foliar spray (Lou et al. 2016; Yuan et al. 2017; Bian et al. 2018). Other studies finding negative effects have applied biochar leachates to soil and have considered a wider range of feedstock types (including wood and manures as well as agricultural residues). Feedstock effects are also confounded in part with biochar type (slow pyrolysis char vs. hydrochar) in these analyses; pronounced negative effects of slow pyrolysis biochar leachates on biomass growth derive from one study (Gale et al. 2016). It is possible that foliar applications of biochar leachates may have positive effects on plant performance as a consequence of bio-toxicity: i.e., by controlling foliar pathogens or herbivore impacts. Dilute wood vinegar has a long history of use as a biocidal agent (Tiilikkala et al. 2010), and wood vinegar constituents are commonly found in biochar leachates (Buss et al. 2015; Rombolà et al. 2015; Gale et al. 2016). An additional recent study indicates positive effects on seedling development of certain compounds volatized from biochar, but not leached compounds (Backer et al. 2018). The claim that organic constituents in biochar leachates are responsible for observed positive effects (e.g., Yuan et al. 2017) is not rigorously supported, in that studies to date have not experimentally isolated potential effects of leached organic compounds vs. inorganic nutrient effects, as has been done in some prior germination studies (e.g., Rogovska et al. 2012). Thus, the current literature does not support either a general positive or negative effect of biochar leachates on plant growth. However, it is clear that additional research is necessary to elucidate whether biochar leachates can have direct positive effects, and whether such effects can be attributed to specific organic constituents.

In summary, available data are sufficient to give some guidance on best practices for biochar post-processing. The most salient points relevant to biochar producers are that heating and aeration treatments are generally preferable to leaching to reduce volatile organics, and that processing biochars to an intermediate particle size (of ~ 0.5–1.0 mm) may generally enhance effectiveness as a soil amendment. However, it is also apparent that there are numerous outstanding research gaps related to biochar post-processing. I conclude with a brief outline of important future research priorities.

### Optimization of particle size or particle size mixtures

Given that milling of biochar to specific size specifications is the single-most promising post-processing technology, further work in this area is a high priority. Biochar particle size effects have recently received considerable attention; however, no prior study on plant growth responses has investigated more than 3 particle size categories. Optimum biochar particle size likely differs with soil particle size, and research that focuses on optimization of biochar particle size for major soil texture classes seems essential. There is also evidence that plants show very strong species-specific responses to biochar particle size (Liao and Thomas 2019); however, it is not clear whether this is a common pattern, or whether this response is predictable from plant soil preferences. Effects of particle size mixtures also deserve attention: both milled and unprocessed biochars typically show a wide distribution of particle sizes, and effects on key soil parameters such as water holding capacity are likely to depend on characteristics of this overall distribution as well as mean biochar particle size.

### Optimization of heating and aeration treatments

Biochar post-processing involving heating and aeration also showed particularly large positive effects (Fig. 1), but these data derive from relatively few studies, and there has been little research on optimizing this process. An important consideration from both an economic perspective, and in developing carbon-negative technologies from a complete life-cycle perspective, is minimization of additional energy inputs. Recycling of heat generated during pyrolysis for this purpose may be feasible, as may use of natural environmental exposure over an extended period. Related to this there is considerable evidence for agronomic benefits of aged biochar; however, natural ageing of biochar has not been directly investigated as a post-production treatment.

### Development of biochar pellets and granules as soil amendments

The meta-analysis results highlight both the lack of trials examining effects of engineered biochar aggregates (biochar granules or pellets) on plant growth, and high variability in results to date (Fig. 1). Optimization of aggregate size is an important consideration that has not been investigated from the point of view of either soil or plant responses. Biochar pellets and granules are likely to have reduced water retention relative to raw biochar, but this has likewise received little attention (Bartocci et al. 2017). Pellets and granules are also likely to have distinct effects, but such differences have also not been investigated. In addition, the main motivation for use of pellets or granules is mitigation of dust exposure and biochar erosion, but measurements on these processes are almost completely lacking.

### Post-processing of biochars generated using alternative pyrolysis technologies

Most work on post-processing has focused on biochars produced by slow pyrolysis, with some attention to leaching studies of hydrochars. Post-processing of fast-pyrolysis biochars, microwave biochars, and high-carbon wood ashes remains largely neglected. Hydrochars are generally recognized to have high concentrations of organic phytotoxic compounds that preclude their use in soil applications (Bargmann et al. 2013; Busch et al. 2013). However, recent results suggest the potential for heat treatment with an inert purging gas to produce advanced hydrochars free from phytotoxicity (Hitzl et al. 2018). Fast-pyrolysis biochars similarly have phytotoxicity issues (Gezahegn et al. 2021), and similar post-production processing strategies might be feasible in this case as well. Biochars produced using microwave technologies have promising properties but have been very little examined in terms of plant responses (Mohamed et al. 2017) and may also require specific post-processing steps. High-carbon wood ash generated from wood gasification facilities may also qualify as a biochar, but commonly displays toxicity due to toxic metals (e.g., Bieser and Thomas 2019). Post-processing steps to enhance carbon content and reduce metal concentrations in high-carbon wood ash would greatly improve the potential range of applications of this material.

### Novel biochar post-processing technologies

Finally, there are a wide variety of promising post-processing techniques for biochar that have received little or no focused research attention. Triboelectric particle sorting is commonly used in the coal industry to produce coal products with enhanced physio-chemical properties (e.g., Dwari and Rao 2009), but there appears to be no investigation of this strategy for biochars. This approach might be preferable to mechanical sieving on an industrial scale, since few moving parts are involved; it may also specifically assist in removal of biochar particles with high metals content. Pervaporation, in which volatile organics are removed by partial vaporization through a selectively permeable membrane (Peng et al. 2003), seems highly applicable to biochar post-processing to reduce sorbed organics of bio-toxicity concern. Finally, prilling, widely used in the fertilizer industry to generate highly uniform spheres (e.g., Rahmanian et al. 2015), has also not been applied to biochar, and would be of particular interest in development of soil amendment products that incorporate biochar in nitrogen fertilizers. Incorporation of biochar particles in prilled urea might specifically enhance retention of applied N in agricultural and forestry applications.

## Supplementary Information

Below is the link to the electronic supplementary material.

Supplementary file1 (TXT 13 KB)

Supplementary file2 (TXT 12 KB)

Supplementary file3 (TXT 4 KB)

Supplementary file4 (DOCX 20 KB)

## Data Availability

Data are available as supplementary information.
