# Supplementary material for: Post-processing of biochars to enhance plant growth responses: a review and meta-analysis
Source: Biochar. 2021 Aug 25;3(4):437–55. doi: 10.1007/s42773-021-00115-0 (PMC8547209; doi:10.1007/s42773-021-00115-0)
Supplement: Supplementary file 4 — Supplementary file4 (DOCX 20 KB) [file 42773_2021_115_MOESM4_ESM.docx]

Supplementary information

Description of primary data files associated with Thomas, S.C. (2021) Post-processing of biochars to enhance plant growth responses: a review and meta–analysis. Biochar x: x-x.

All files are formatted as tab-delimited text files.

Files and brief description:

Biochar_postprocessing_file1_processtype.txt

Compiled meta-analysis data corresponding to Fig. 1, showing growth responses to biochar post-processing modifications.

Biochar_postprocessing_file2_particlesize.txt

Compiled meta-analysis data corresponding to Fig. 2, showing plant growth responses to variation in biochar particle size.

Biochar_postprocessing_file3_leachates.txt

Compiled meta-analysis data corresponding to Fig. 3, showing plant growth responses to additions of biochar leachates

Detailed description of data fields:

File: Biochar_postprocessing_file1_processtype.txt

| studynum | Study number |
| --- | --- |
| citation | Citation corresponding to reference list in paper |
| location | Country in which study took place |
| modtype | Modification type (classified as “activation” for steam or chemical activation, “particle” for particle size manipulation, “leaching” for water washing or leaching, “heat” for heat treatment, and “pellet” for pelletization or granulation) |
| data | Source of data (classified as “table” for values copied from tables, “graph” for values derived from digitization of graphs, and “orig” where the original raw data were utilized) |
| Exptype | Type of experiment (classified as “pot” for pot trials, “field” for field trials, and “hydroponic” for hydroponic cultivation) |
| plant | Latin name for species of plant examined |
| measure | Growth measure analyzed (“biomass” for total plant biomass, “ag biomass” for aboveground biomass, “yield” for agronomic yield measure, “fresh mass” for fresh (undried) mass, and “fruit mass” for mass of fruit) |
| units | Unit of measurement (“g” = grams; kg ha-1 = kilogram per hectare; g m-2 = gram per square meter; “count” = numerical count; “mg” = milligram; “%” = percent) |
| imputsd | Was the standard deviation of the data imputed from other data as described in methods (yes / no) |
| reps | Number of replicates per treatment in the study |
| days | Length of growth period of study in days |
| control | Value of measure for untreated control (in original units in paper) |
| sdc | Standard deviation for untreated control (in original units in paper) |
| bc | Value of measure for unprocessed biochar (in original units in paper) |
| sdbc | Standard deviation for unprocessed biochar (in original units in paper) |
| bcmod | Value of measure for processed biochar (in original units in paper) |
| sdbm | Standard deviation for processed biochar (in original units in paper) |

File: Biochar_postprocessing_file2_particlesize.txt

| studynum | Study number |
| --- | --- |
| citation | Citation corresponding to reference list in paper |
| location | Country in which study took place |
| mod | Modification type (classified as “sieving” for mechanical sieving, “grinding” for grinding (often followed by sieving), and “nanoextract” for processes to extract nano particles) |
| partcat | Particle size category (1 = <0.1 mm; 2 = 0.1 – 0.49 mm; 3 = 0.50 – 0.99 mm; 4 = 1.00 – 1.99 mm; 5 = 2.0 – 3.9 mm; 6 = >4.0 mm) |
| data | Source of data (classified as “table” for values copied from tables, “graph” for values derived from digitization of graphs, and “orig” where the original raw data were utilized) |
| exptype | Type of experiment (classified as “pot” for pot trials, “field” for field trials, and “hydroponic” for hydroponic cultivation) |
| plant | Latin name for species of plant examined |
| measure | Growth measure analyzed (“biomass” for total plant biomass, “ag biomass” for aboveground biomass, “yield” for agronomic yield measure, “fresh mass” for fresh (undried) mass, and “fruit mass” for mass of fruit) |
| units | Unit of measurement (“g” = grams; kg ha-1 = kilogram per hectare; “count” = numerical count; “mg” = milligram) |
| imputsd | Was the standard deviation of the data imputed from other data as described in methods (yes / no) |
| reps | Number of replicates per treatment in the study |
| days | Length of growth period of study in days |
| control | Value of measure for untreated control (in original units in paper) |
| sdc | Standard deviation for untreated control (in original units in paper) |
| bc | Value of measure for unprocessed biochar (in original units in paper) |
| sdbc | Standard deviation for unprocessed biochar (in original units in paper) |
| bcmod | Value of measure for processed biochar (in original units in paper) |
| sdbm | Standard deviation for processed biochar (in original units in paper) |

File: Biochar_postprocessing_file3_leachates.txt

| studynum | Study number |
| --- | --- |
| citation | Citation corresponding to reference list in paper |
| location | Country in which study took place |
| feedstock | Biochar feedstock used (description as given in original publication) |
| Feedtype | Feedstock type (classified as “manure” for manures, “agric” for agricultural residues, and “wood” for woods) |
| bctype | Biochar type (classified as “slow” for slow pyrolysis biochar, and “hydro” for hydrochar) |
| temp | Pyrolysis temperature (°C) (“NA” = not available) |
| application | Biochar leachate application method (description as given in original publication) |
| apptype | Biochar leachate application method type (classified as “soil” for soil applications, and “surface” for surface applications to soil surface or foliage) |
| data | Source of data (classified as “table” for values copied from tables, “graph” for values derived from digitization of graphs) |
| exptype | Type of experiment (classified as “pot” for pot trials) |
| plant | Latin name for species of plant examined |
| measure | Growth measure analyzed (“biomass” for total plant biomass, “fresh mass” for fresh (undried) mass) |
| units | Unit of measurement (“g” = grams; “mg” = milligram) |
| imputsd | Was the standard deviation of the data imputed from other data as described in methods (yes / no) |
| reps | Number of replicates per treatment in the study |
| days | Length of growth period of study in days (NA = not available) |
| control | Value of measure for untreated control (in original units in paper) |
| sdc | Standard deviation for untreated control (in original units in paper) |
| bc | Value of measure for biochar leachate treatment (in original units in paper) |
| sdbc | Standard deviation for biochar leachate treatment (in original units in paper) |
